# The Dispersion of Pulp-Fiber in High-Density Polyethylene via Different Fabrication Processes

**DOI:** 10.3390/polym10020122

**Published:** 2018-01-26

**Authors:** Xiaohui Yang, Guangzhao Wang, Menghe Miao, Jinquan Yue, Jianxiu Hao, Weihong Wang

**Affiliations:** 1 Key Lab of Bio-based Material Science Technology of Education Ministry, Northeast Forestry University, Harbin 150040, China; xiaohuiyang90@163.com (X.Y.); guangzhaowang1@163.com (G.W.); yuejinq@163.com (J.Y.); jianxiuhao2016@163.com (J.H.); 2 CSIRO Manufacturing, 75 Pigdons Road, Waurn Ponds, VIC 3216, Australia; Menghe.Miao@csiro.au

**Keywords:** pulp fiber, polyethylene, composites, fiber dispersion, drying

## Abstract

In this study, a pulp beating machine was used to premix the pulp fibers with high density polyethylene (HDPE) particles in water. The wet or pre-dried pulp fiber/HDPE mixture was then melt-compounded by a twin screw extruder. For further improving the dispersion of pulp fiber, some mixture was forced to pass through the twin-screw extruder twice. The resulting mixture was compression molded to the composite. The fiber distribution was observed by the aid of an optic and scanning electron microscope. The mechanical and rheological properties and creep resistance of the composites were characterized. Test results demonstrate that when the wet pulp fiber/HDPE mixture was subjected to pre-pressing and oven drying prior to extrusion compounding, the resulting composites exhibited homogeneous fiber distribution, superior flexural property, creep-resistance, and high storage modulus. Particularly, its flexural strength and modulus were 57% and 222% higher, respectively, than that of the neat HDPE, while the composites prepared without pre-dried were 19% and 100% higher, respectively. Drying the wet mixture in advance is more effective than re-passing through the extruder for improving the fiber dispersion and composite performance.

## 1. Introduction

Natural fibers—Such as hemp, wood, or bamboo—Possess great potential to replace conventional synthetic fibers (i.e., glass fiber, carbon fiber, etc.) for petroleum-based polymer composites due to their renewability, biodegradability, low density, as well as the high specific mechanical properties [1,2,3]. As a product of natural fibers, pulp fibers have an average elastic modulus of 40 GPa, which is higher than bulk natural fibers (10 GPa) [4]. Pulp fibers are typically finer and more uniform than natural fibers, so it is possible that there are fewer defects in the composites and it has potentially better performance [5]. In addition, the discarded polyolefin and paper have become an important part of the waste stream. Reinforcing polyolefin with paper fibers would be a good choice to recycle these materials.

Commonly, pulp/thermoplastic composites are prepared by a process consisting of mixing, melt-compounding, and extrusion or injection. Several options are available for the melt-compounding of pulp/thermoplastic, including using a twin-screw extruder [6,7], internal mixer [8,9], or multi-kinetic mixer [10]. In most cases, the pulp fibers were dried before mixing and melt-compounding [11,12,13]. However, the dried natural fibers in general present low bulk density. A large difference in bulk densities between pulp fibers and polymer matrix may cause problems in the mixing, such as poor feeding, bridging, surging, and finally non-uniform dispersion [14,15]. Valente et al. [16] also reported that it is difficult to mix fluffy paper fibers with polymers that have high viscosity. Owing to the fact that fibers agglomerate at 5.5 wt %, a high weight percentage of paper fibers cannot be introduced into an extruder. The dispersion of pulp fibers in polymer matrix is a difficult task for pulp/thermoplastic composite fabrication.

Since pulp fibers can be easily separated in water attributable to the polarity of cellulose and are usually obtained as an aqueous suspension, mixing pulp with thermoplastics component in water is expected to be an effective approach [17]. However, the liquid medium in pulp/polymer mixture still needs to be removed before melt-compounding. During the drying process, the contraction of the fibrils pulls the fibers closer together, promoting the formation of hydrogen bonds between the pulp fibers [18,19], and leading to fiber re-agglomeration. Some methods have been investigated to solve this problem. For example, Suzuki et al. [20,21] kneaded refiner-treated never-dried kraft pulp with powdered polypropylene by a twin-screw extruder at 0 °C. The wet pulp/polymer mixture was then directly melt-extruded. By the aid of coupling agents, the mechanical properties of the resulting composites were improved. Other research shifted to compression molding. Du [5] and Thumm et al. [22] prepared a pulp/polymer mixture in the form of mats after dewatering and followed by oven-drying. The mats were thermoformed into composites in a match mold.

Another critical factor determining the mechanical properties of composites is the interphase between reinforcing element and matrix. However, the compatibility between the hydrophilic cellulose fibers and the hydrophobic matrix—Especially hydrocarbon matrixes such as polypropylene (PP) and polyethylene (PE)—is poor [23]. The design of an appropriate interphase for efficient stress-transfer from the matrix to the reinforcements is required [24]. Adding a coupling agent is an effective method. Maleated polyolefins (MAPP, MAPE, and so on) are the most common coupling agents used to improve the natural fiber–matrix interface via the chemical interactions of –OH/maleic [25]. Meanwhile, surface treatment with carbon nanotubes (CNTs) [26], surfactants [27], or chemical modification [28] can also help disperse the natural fibers within the matrix.

In the present study, MAPE was used to improve the compatibility between the pulp fiber and high density polyethylene (HDPE) matrix. New methods of dispersing pulp fiber in HDPE matrix were developed for the preparation of pulp fiber/HDPE composites. The pulp fibers and HDPE powder were premixed in water. After dewatering, the wet, oven-dried, or prepressed oven-dried pulp fiber/HDPE mixtures were melt-compounded by a twin-screw extruder. To further improve the dispersion of pulp fiber in HDPE matrix, part of the pulp fiber/HDPE mixtures was forced to pass through the twin-screw extruder twice. The effects of these methods on the dispersion of pulp fiber in HDPE and the properties of the resulted composites are discussed.

## 2. Materials and Methods

### 2.1. Materials

Bleached hardwood pulp board was supplied by Mudanjiang Hengfeng Paper Co., Ltd. (Mudanjiang, China). The length, width and aspect ratio of pulp fiber were 1.0 mm, 20.3 μm, and 49.3, respectively. HDPE (grade: 5000 s; melt flow index: 0.8–1.1 g/10 min at 190 °C; density: 0.949–0.953 g/cm^3^) was purchased from Petro China Daqing Petrochemical Company (Daqing, China). MAPE (grafting percentage: 0.9%) was obtained from Shanghai Sunny New Technology Development Co., Ltd. (Shanghai, China). Prior to mixing with pulp fibers, HDPE and MAPE granules were milled into a powder (80–120 mesh) by using a mill (FZ102, TAISITE, Tianjin, China).

### 2.2. Dispersion of Pulp Fiber and Preparation of Composites

The pulp board was immersed in water for 24 h and then transferred to a pulping beater (ZQS2-23, Shanxi University of Science and Technology Machinery Factory, Xian, China). HDPE and MAPE powders were added in the beater. The mixture was disintegrated until the fibers were fully dispersed and uniformly mixed with the plastic powders. Following, the pulp fiber/HDPE slurry was dewatered by using a spin-dryer (TT75-S189C, Littleswan, Wuxi, China) until the water content of the mixture decreased to ~50%.

**Figure 1:**
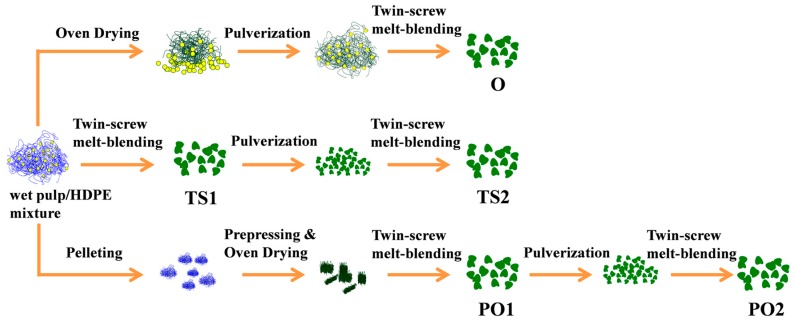
Fabrication processes of pulp fiber/HDPE mixture. Mixture O: wet pulp fiber/HDPE mixture was dried by oven. Mixture TS1: wet pulp fiber/HDPE mixture was dried by twin-screw extruder. Mixture TS2: TS1 was melt-extruded again. Mixture PO1: wet pulp fiber/HDPE mixture was dried by prepressing and oven. Mixture PO2: PO1 was melt-extruded again.

To remove the residual water, the wet pulp fiber/HDPE mixture experienced oven drying (O), twin-screw extruding (TS), or prepressing and oven drying (PO). The mixture was forced to re-pass through the twin-screw extruder to further improve the dispersion of the pulp fiber in the HDPE matrix melt-compounding in the twin-screw was conducted at the speed of 40 rpm at 145–165 °C. As shown in Figure 1, different fabrication routes prepared pulp fiber/HDPE compound mixtures. The weight ratio of pulp fiber/ HDPE /MAPE was 50/47/3.

Method (a): the wet pulp fiber/HDPE mixture was dried out at 80 °C in an oven. After drying, the separated fibers were tightly regrouped into blocks. The dry blocks were scattered into particles by using a pulverizer (HNEB-115K, TAISITE, Tianjin, China). These particles were manually forced into a twin-screw extruder (Nanjing Rubber and Plastics Machinery Plant, Nanjing, China) for compounding. The resulting mixture was named O.

Method (b): without prior drying, the wet pulp fiber/HDPE mixture was manually forced into the twin-screw extruder, and then melt-compounded. Water in the mixture was expected to evaporate during compounding. The resulting mixture was named TS1.

Method (c): part of mixture TS1 was pulverized into small particles by using the above-mentioned pulverizer and fed into the twin-screw extruder again. The resulting mixture was named TS2.

Method (d): the wet pulp fiber/HDPE mixture was manually torn into small pieces (diameter: ~8 mm). These pieces were pressed into slices with a thickness of 3 mm under 10 MPa at 105 °C for 5 min. The slices were then oven-dried at 80 °C. The dried mixture slices were fed into the extruder. By this method, the dried mixture can be smoothly fed into the twin-screw extruder without enforcement. The resulting mixture was named PO1.

Method (e): part of mixture PO1 was pulverized into small particles and fed into the twin-screw extruder again. The resulting mixture was named PO2.

The five pulp fiber/HDPE mixtures from the above routes were reduced to small particles by using the pulverizer mentioned above. These particles were compression molded at 165 °C and 15 MPa for 10 min to obtain 4 mm thick composite sheets. The composites were named O, TS1, TS2, PO1, and PO2, respectively corresponding to the mixtures. For comparison, the pure HDPE sheet was also prepared at the same compression molding conditions.

### 2.3. Mechanical Property Test

Samples were stored at 50% RH at room temperature for 48 h before being tested. Mechanical property measurements were performed on specimens at room temperature (relative humidity: ~50%). At least six specimens were tested from each sample.

Flexural tests were performed in accordance with ASTM D 790-03, “Standard Test Methods for Flexural Properties of Unreinforced and Reinforced Plastics and Electrical Insulating Materials”, on 80 × 13 × 4 mm (span length: 64 mm) specimens. A loading speed of 2 mm/min was used during testing.

The unnotched impact strength was determined in accordance with a Chinese standard GB/T 1043.1-2008 (Plastics, Determination of Charpy Impact Properties, Part 1: Non-instrumented Impact Test). A striking velocity of 2.9 m/s and pendulum energy of 2 J were employed to test the specimens, which had dimensions of 80 × 10 × 4 mm (span length: 60 mm).

### 2.4. Morphological Analysis

The quantification of the pulp fiber macro-dispersion in the HDPE matrix was performed by optical image. The thickness of sample was 0.5 mm. The state of dispersion was determined by analyzing the remaining, white appearing fiber agglomerates in the recorded image. The agglomerate area ratio was calculated by the Image J software (NIH, Bethesda, MD, USA). At least five individual recorded images were used for the calculations.

The fiber length distributions and damage assessments of fibers in composite samples were determined after extraction with xylene. The images of the fibers were captured by a light optical microscopy and the fiber length was measured by the Image J software. At least 500 individual recorded fibers were measured for one sample.

A scanning electron microscope (SEM, FEI Quanta 200, Hillsboro, OR, USA) was used to characterize the morphology of paper pulp/HDPE composite. The cross sections of the samples were sputter-coated with gold and subsequently observed in SEM.

### 2.5. Dynamic Rheological Properties

An AR2000ex rheometer (TA Instruments, New Castle, DE, USA) was used to determine the rheological properties of the pulp fiber/HDPE composite. A 25 × 3 mm (diameter × thickness) sample was clamped using a pair of parallel stainless plates (diameter: 25 mm), separated by a target working distance of 3 mm. The test was run at 180 °C and a frequency sweep pattern of 1–600 rad/s.

### 2.6. Short-Time Creep-Recovery Test

The short-time creep-recovery test of rectangular 52 × 10 × 3 mm composite samples was performed in torsion mode using the rheometer mentioned above. The samples were mounted on the sample holder (span: 40 mm), and the chamber temperature was maintained at 30 °C during testing. An isothermal creep test was run under 0.5 MPa for 30 min. The sample was then unloaded, and the strain (of the sample) was continuously recorded for 30 min.

### 2.7. Statistics

Significant differences (α = 0.05) between the different methodologies were determined via analysis of the variance.

## 3. Results and Discussion

### 3.1. Morphology of the Composites

**Figure 2:**
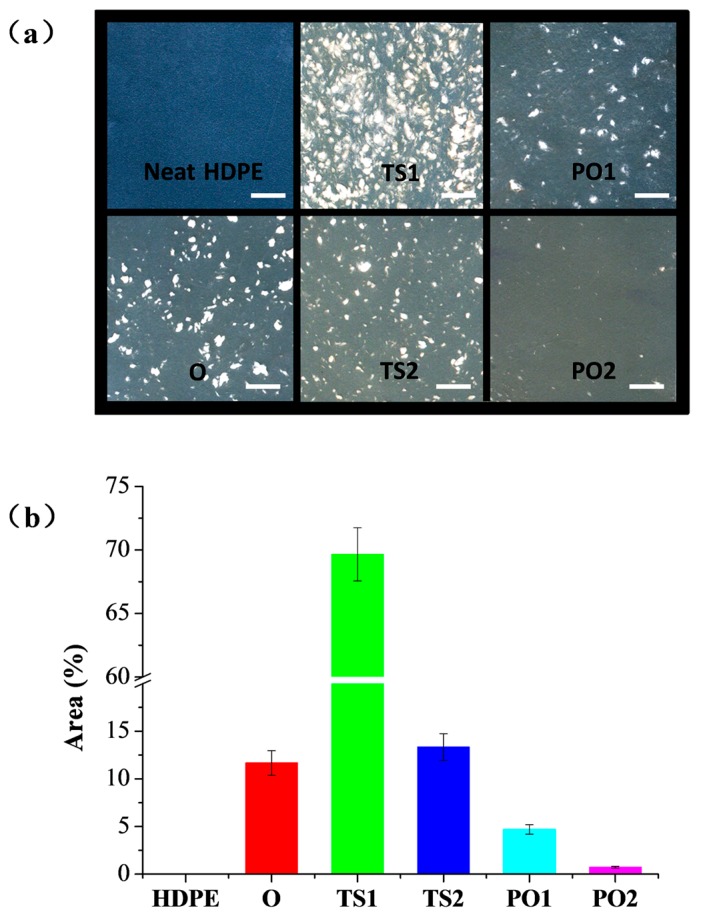
(**a**) Optic photos of neat HDPE and pulp fiber/HDPE composites. The thickness of sample is 0.5 mm. Scale bar was 5 mm. (**b**) Agglomerate area ratio (abbreviated as *A*_A_) of each sample. Composite O: wet pulp fiber/HDPE mixture was dried by oven. Composite TS1: wet pulp fiber/HDPE mixture was dried by twin-screw extruder. Composite TS2: mixture TS1 was melt-extruded again. Composite PO1: wet pulp fiber/HDPE mixture was dried by prepressing and oven. Composite PO2: mixture PO1 was melt-extruded again.

As shown in Figure 2, the composite TS1 showed significant agglomeration of fibers. The agglomerate area ratio (abbreviated as *A*_A_) of composite TS1 was 69.6%. After dewatering, the wet pulp fibers formed a loose block wrapped with HDPE powders. When the wet mixture experienced extruding at high temperature in the twin-screw, water in pulp fibers quickly evaporated. The water evaporation promoted the formation of direct hydrogen bonds between the fibers, which resulted in fiber agglomeration. In addition, water evaporation might form a water vapor layer on the surface of pulp fiber, serving as a protection from shear effect during melt compound [29]. This water vapor layer also slowed down HDPE melting and penetrating into pulp fibers during the entire extrusion process. Therefore, the composite TS1 exhibited extremely poor fiber distribution.

When the wet pulp fiber/HDPE mixture was oven dried prior to the extrusion of compound, most HDPE particles were embedded in pulp fibers. This is beneficial to prevent the formation of hydrogen bond between the pulp fibers. On the other hand, the dried pulp fibers are more brittle than wet fibers, which is advantageous for the dispersion of fibers in matrix. Thus, the resulting composite (i.e., O) exhibits a better distribution of fibers than TS1 (Figure 2). The *A*_A_ of composite decreased from 69.6% to 11.7%. However, during oven drying, some HDPE powders detached from the fibers.

For composite PO1, the wet pulp fiber/HDPE mixture was reduced into small pieces and then experienced hot pre-pressing and oven drying. Pre-pressing embedded HDPE powders tightly between the pulp fibers, and few HDPE powders detached from the mixture during the subsequent oven drying process. Thus, pulp fibers were effectively separated by HDPE particles. This should be the main reason for composite PO1 showing better fiber distribution than composite O. The *A*_A_ of composite decreased from 11.7% to 4.7%.

The composites TS2 (*A*_A_ = 13.3%) and PO2 (*A*_A_ = 0.7%) present less fiber agglomeration than TS1 (*A*_A_ = 69.6%) and PO1 (*A*_A_ = 4.7%), respectively (Figure 2). This indicates that the dispersion of pulp fiber in HDPE could be further improved if the mixture passed through the twin-screw and pulverizer twice. However, even though the composite TS2 were blended twice through the twin-screw, the composite TS2 exhibited a poorer fiber distribution than composite PO1. This indicates that pre-drying is more effective than repeated pulverization and extrusion compounding for fiber dispersion.

**Figure 3:**
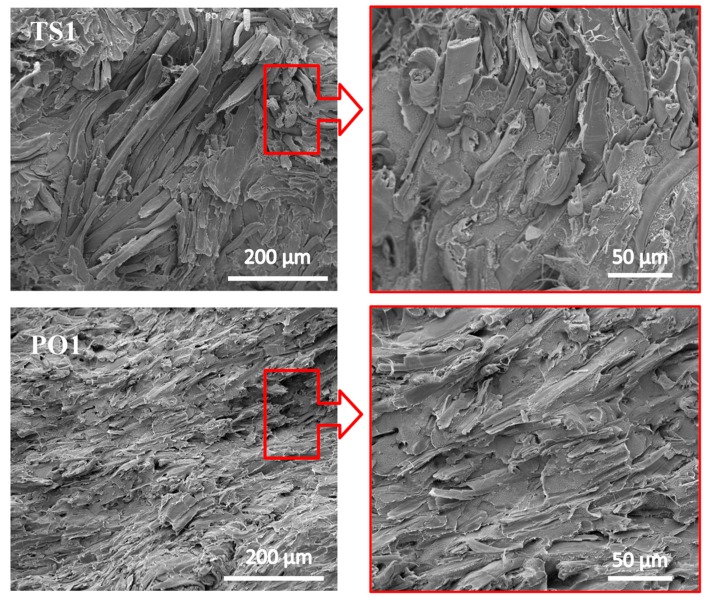
SEM images of the cross section of pulp fiber/HDPE composites. The right image corresponds to the circled region of left image. Composite TS1: wet pulp fiber/HDPE mixture was dried by twin-screw extruder. Composite PO1: wet pulp fiber/HDPE mixture was dried by prepressing and oven.

The SEM images in Figure 3 show that HDPE does not penetrate into agglomerate fibers in TS1. Most fibers inside lack adhesion or separation, only fibers on the agglomeration contact the matrix. These locations weaken the stress transmission. In contrast, evenly dispersed fibers in matrix in composite PO1 possess larger interaction area in matrix than those of agglomerated fibers, which is more effective for stress transfer from the matrix to the reinforcing elements.

### 3.2. Fiber Damage

**Figure 4:**
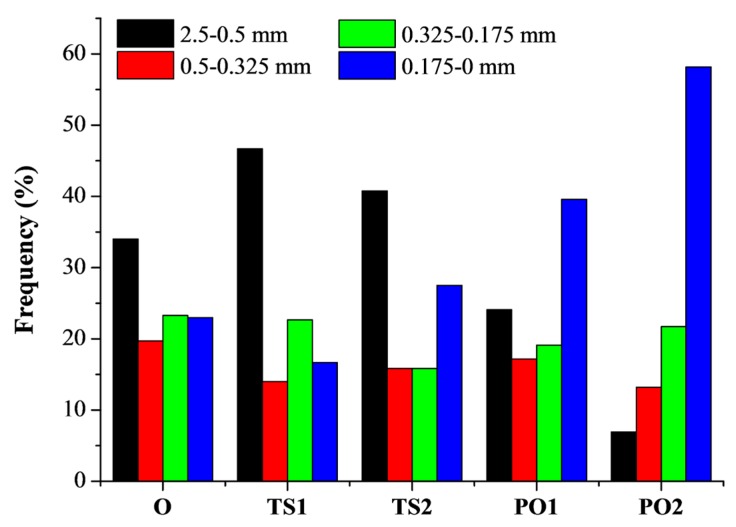
Fiber length distribution of fibers extracted from pulp fiber/HDPE composites. Composite O: wet pulp fiber/HDPE mixture was dried by oven. Composite TS1: wet pulp fiber/HDPE mixture was dried by twin-screw extruder. Composite TS2: mixture TS1 was melt-extruded again. Composite PO1: wet pulp fiber/HDPE mixture was dried by prepressing and oven. Composite PO2: mixture PO1 was melt-extruded again.

The effect of composite fabrication methods on fiber damage was examined. The length distribution of fibers extracted from pulp fiber/HDPE composites fabricated by different methods is significantly changed (Figure 4). It reveals that composite TS1 contains more fibers above 0.5 mm (46.7%) compared to O (34.0%) and PO1 (24.1%). This suggests that the wet pulp fiber/HDPE mixture reduced fiber broken during twin-screw extrusion due to better plasticity, while dry fibers are fragile and are easily sheared. Fibers less than 0.175 mm accounted for 58.2% of PO2, which is most among these tested methods. This result is closely related with the fiber distribution. The good fiber dispersion implies serious fiber damage.

### 3.3. Mechanical Properties of the Composites

**Figure 5:**
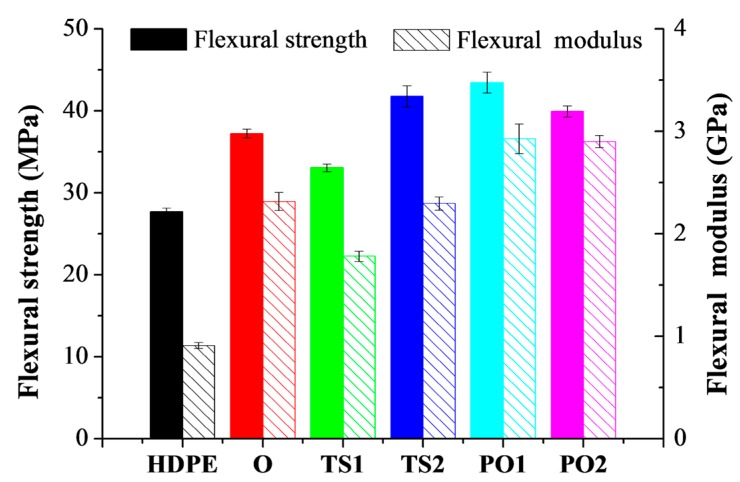
Flexural strength and modulus of pulp fiber/HDPE composites. Composite O: wet pulp fiber/HDPE mixture was dried by oven. Composite TS1: wet pulp fiber/HDPE mixture was dried by twin-screw extruder. Composite TS2: mixture TS1 was melt-extruded again. Composite PO1: wet pulp fiber/HDPE mixture was dried by prepressing and oven. Composite PO2: mixture PO1 was melt-extruded again.

Figure 5 and Table 1 show that the flexural property of HDPE was significantly improved when HDPE was filled with pulp fibers. The flexural strength and modulus of composites manufactured via different drying methods were in the following order: PO1 > O > TS1, which is consistent with the dispersion homogeneity of the fiber in the composite (Figure 2). The composite PO1 displayed the highest flexural strength and modulus, 57% and 222% higher, respectively, than that of the neat HDPE, while TS1 exhibited 19% and 100% higher, respectively. Theoretically, the fiber damage reduces the reinforcement effect of the fibers. Nevertheless, it has also been reported that good dispersion of the fibers could improve the mechanical properties of the composite, though simultaneously reducing the fiber length [30,31]. As shown in Figure 3 and Figure 4, the pulp fibers in PO1 were damaged more severely than in O and TS1, though they were evenly distributed throughout the HDPE matrix with good fiber/polymer adhesion, assisting in the efficient transfer of force from the matrix to the fiber.

The flexural strength and modulus of TS2 were significantly higher than those of TS1 (Figure 5). Though both of them were prepared from the un-dried pulp fiber/HDPE mixture before extrusion, the former was subjected to extrusion compounding twice, resulting in the improvement of fiber distribution and adhesion to the matrix. However, the flexural strength of PO2 (39.9 MPa) was lower than that of PO1 (43.4 MPa). The process of pre-pressing, oven drying, plus twin-screw extruding (PO1) has dispersed fibers suitably in HDPE. Owing to the shear forces acting on the materials, these fibers were further damaged after two rounds of extrusion and pulverization.

Fiber drying, mixing, and compounding with HDPE occurred simultaneously during extrusion for TS, which significantly increased the production efficiency of composite preparation. Compared to composite O (37.2 MPa), TS2 displayed higher flexural strength (41.8 MPa), suggesting that the screw drying can be used to replace the time-consuming oven drying for pulp/HDPE composite fabrication.

**Figure 6:**
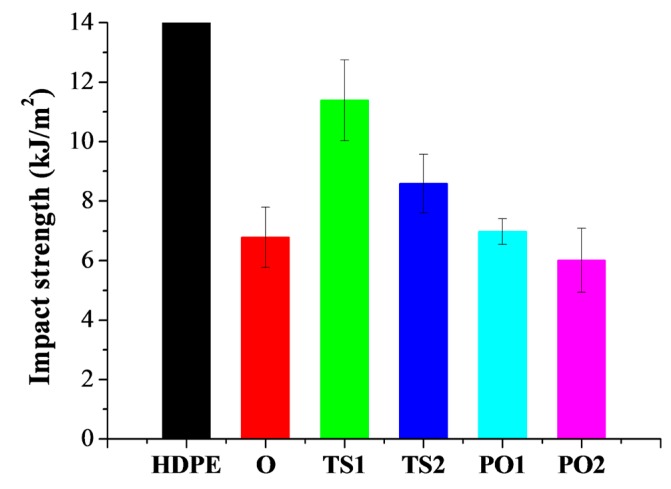
Impact strength of pulp fiber/HDPE composites. The neat HDPE was unbroken. Composite O: wet pulp fiber/HDPE mixture was dried by oven. Composite TS1: wet pulp fiber/HDPE mixture was dried by twin-screw extruder. Composite TS2: mixture TS1 was melt-extruded again. Composite PO1: wet pulp fiber/HDPE mixture was dried by prepressing and oven. Composite PO2: mixture PO1 was melt-extruded again.

Compared to neat HDPE, all pulp fiber reinforced HDPE decreased in impact strength (Figure 6), which is adverse to flexural properties. In other words, pulping fiber reinforcement stiffens the HDPE matrix, but decreases its toughness. This is in accordance with the results reported in other research [32,33]. The impact strength of TS1 was the highest among these composites (Figure 6 and Table 1). This may be due to its poor distribution of pulp fibers and weak bonding between pulp fibers and HDPE matrix, as shown in Figure 3. Voids existed in aggregated fiber bundles. However, these defects could absorb impact energy. The impact strength of TS2 and PO2 was lower than that of TS1 and PO1, respectively. This is in accordance with the fiber damage. The number of stress concentration points increases with the fiber damage, which leads to a decrease in the unnotched impact strength.

### 3.4. Dynamic Rheological Properties of the Composites

**Figure 7:**
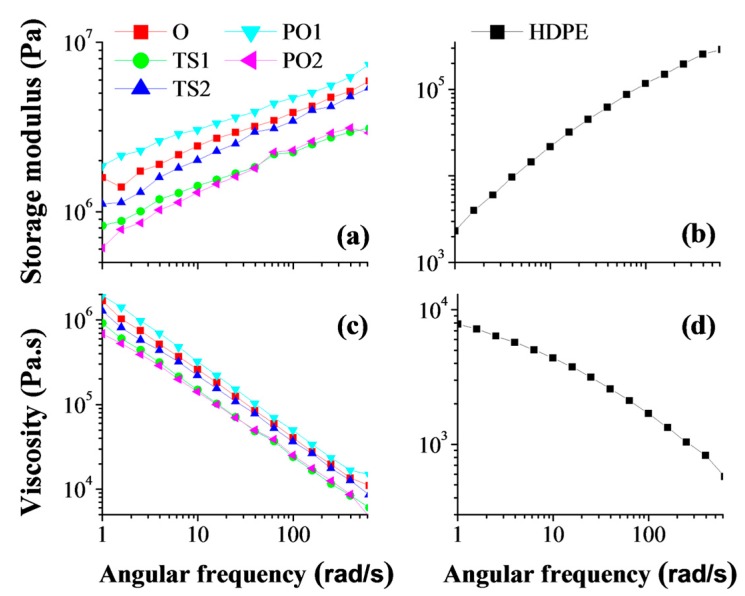
Storage modulus of (**a**) pulp fiber/HDPE composites and (**b**) neat HDPE; Complex viscosity of (**c**) pulp fiber/HDPE composites and (**d**) neat HDPE. Composite O: wet pulp fiber/HDPE mixture was dried by oven. Composite TS1: wet pulp fiber/HDPE mixture was dried by twin-screw extruder. Composite TS2: mixture TS1 was melt-extruded again. Composite PO1: wet pulp fiber/HDPE mixture was dried by prepressing and oven. Composite PO2: mixture PO1 was melt-extruded again.

The storage modulus of all samples increased linearly with the angular frequency. Changing the frequency had a greater effect on the neat HDPE than on the composites. The storage modulus of the pulp fiber/HDPE composites was considerably higher than that of the neat HDPE (Figure 7). The increase of storage modulus means the system changed from viscous to more elastic. This effect may be attributed to the mechanical reinforcement of the percolated filler network in the blend systems. The micro-scale filler had an impact similar to the nano-scale filler the melt-rheology on a polymer thermoplastic matrix. As shown in the study done by Liebscher et al. [34], the addition of CNTs to the blend system caused a noticeable increase of storage modulus at lower frequencies.

The complex viscosity of both neat HDPE and pulp fiber/HDPE composites decreased with the increase of angular frequency, signifying that a shear thinning process occurred [35]. Changing the frequency had a greater effect on the composites than on the neat HDPE (Figure 7). This may be attributed to the more significant non-Newtonian pseudoplastic fluid characteristics of the composites. Owing to interaction between the rigid fibers, the movement of the polymer matrix in the fiber network is more difficult than in neat polymer [36,37].

The storage modulus and complex viscosity of PO1 were higher than that of composites O and TS1; this is possibly owing to the relatively even dispersion and better compounding. The evenly dispersed fibers possessed a larger interaction area in matrix than the agglomerated fibers, and the evenly dispersed fibers formed a more effective adhesion with HDPE matrix. Therefore, it is more difficult for the melt polymer to move between the evenly dispersed fibers, resulting in higher modulus.

Reprocessing also played an important role in the rheological property of the composite. The storage modulus and complex viscosities of PO1 were higher than that of PO2, respectively. The fibers in PO2 underwent more severe damage caused by the twice pulverization and melt-extruding than those in PO1. Furthermore, the longer fibers in PO1 may form more stable and complex entanglements, potentially hindering the motion of the HDPE chain. The interaction between shorter fibers (PO2) was more easily disrupted than that between longer fibers (PO1), thus allowing relatively free motion of the polymer chain and leading the former to a lower storage modulus and smaller complex viscosity.

On the contrary, the storage modulus and complex viscosity of TS2 were higher than that of TS1. The mixture of TS2 experienced two passes of extrusion with significantly reduced fiber agglomerates. Though the pulp fibers were also damaged when subjected to the second extrusion, the uniform fiber distribution positively contributed more than fiber damage to the properties of TS2. Similar to PO1, the uniformly dispersed fibers in TS2 had larger contacting surface area with the matrix, resulting in higher shear viscosity and storage modulus.

### 3.5. Creep-Recovery Test of the Composites

**Figure 8:**
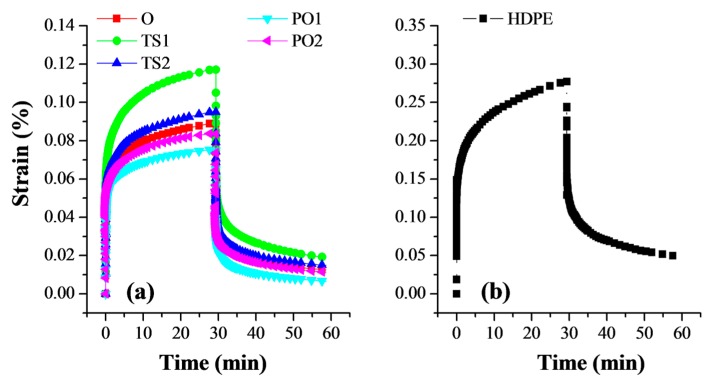
Creep-recovery curves of (**a**) pulp fiber/HDPE composites and (**b**) neat HDPE. Composite O: wet pulp fiber/HDPE mixture was dried by oven. Composite TS1: wet pulp fiber/HDPE mixture was dried by twin-screw extruder. Composite TS2: mixture TS1 was melt-extruded again. Composite PO1: wet pulp fiber/HDPE mixture was dried by prepressing and oven. Composite PO2: mixture PO1 was melt-extruded again.

Creep is the continuous accumulation of deflection over time when the material is subjected to a constant load [38]. The creep resistance is an important property of material. The large creep and residual deformation of material indicate poor creep resistance. The neat HDPE exhibits larger creep and residual deformation than the pulp fiber/HDPE composites (Figure 8). The creep-recovery of pulp fiber/HDPE composites varied between the fabrication methods and was consistent with their flexural property. The composites with higher flexural modulus presented better resistance to creep. The creep and residual deformation of composite PO were significantly lower than that of composites O and TS. This is owing to the fact that they had tight adhesion between pulp fibers and HDPE matrix and exhibited uniform fiber dispersion, limiting the motion of matrix molecules and reduced defect points caused by the lack of matrix penetration. In un-reinforced locations, the matrix behaved like pure HDPE. Thus, the composite TS1 showed the highest strain and the lowest recovery among the five composites, which is attributable to its poor fiber dispersion.

## 4. Conclusions

In this study, several methods for dispersing pulp fibers in HDPE matrix were investigated. It was found that when the wet pulp fiber/HDPE mixture was directly fed into a twin-screw extruder, the resulting composite had significantly improved flexural properties, storage modulus, and the resistance to creep over neat HDPE. A second pass through the extruder and pulverizer further improved the fiber dispersion and flexural properties. Moreover, drying before extrusion was more effective than repassing through the extruder. The best drying method was pre-pressing followed by oven drying, which resulted in composites with the most uniform fiber dispersion, the highest flexural property and storage modulus, and the best resistance to creep. Using this method, the compound can be fed into the extruder without enforcement.

## Figures and Tables

**Table 1:** Results of the analysis of variance for mechanical properties of pulp fiber/HDPE composites.

Composites	Flexural strength (MPa)	Flexural modulus (GPa)	Impact strength (kJ/m^2^)
O	37.2 ± 0.5 ^a,b^	2.3 ± 0.09 ^a,b^	6.8 ± 1.0 ^a,b^
TS1	33.0 ± 0.5 ^a,b,c^	1.8 ± 0.05 ^a,b,c^	11.4 ± 1.4 ^a,b,c^
TS2	41.8 ± 1.3 ^a,c^	2.3 ± 0.06 ^a,c^	8.6 ± 1.0 ^a,c^
PO1	43.4 ± 1.3 ^a,b,d^	2.9 ± 0.14 ^a,b,e^	7.0 ± 0.5 ^a,b,d^
PO2	39.9 ± 0.7 ^a,d^	2.9 ± 0.06 ^a,e^	6.0 ± 1.1 ^a,d^

^a^ Significant for varying fabrication methods; ^b^ Significant for varying drying methods; ^c^ Significant for the number of times passed through the twin-screw extruder for TS; ^d^ Significant for the number of times passed through the twin-screw extruder for PO; ^e^ Not significant for the number of times passed through the twin-screw extruder for PO.
